# Posterior reversible encephalopathy as the first manifestation of Bickerstaff’s brainstem encephalitis

**DOI:** 10.1186/s12883-016-0737-6

**Published:** 2016-11-08

**Authors:** Pei-Ru Chen, Shih-Pin Chen

**Affiliations:** 1 Department of Neurology, Neurological Institute, Taipei Veterans General Hospital, Taipei, 112 Taiwan; 2 Faculty of Medicine, National Yang-Ming University School of Medicine, Taipei, Taiwan; 3 Institute of Clinical Medicine, National Yang-Ming University, Taipei, Taiwan

**Keywords:** Bickerstaff’s brainstem encephalitis, Guillain-Barre syndrome, Miller-Fisher syndrome, Posterior reversible encephalopathy syndrome

## Abstract

**Background:**

Posterior reversible encephalopathy syndrome (PRES) has been associated with Guillain-Barre syndrome in rare cases. Here we report a patient in whom PRES was the presenting manifestation of Bickerstaff’s brainstem encephalitis.

**Case presentation:**

A 75-year-old woman presented with acute onset of hypertension, headache, blurred vision, and left eyelid drooping. Magnetic resonance imaging of the brain showed characteristic PRES lesions involving the parietal and occipital lobes bilaterally. On the 6th day after symptom onset, the patient developed complete ptosis and external ophthalmoplegia of both eyes, progressive ataxia, and bilateral lower limb weakness. Cerebrospinal fluid analyses revealed albuminocytological dissociation (protein: 66.6 mg/dL, WBC: 0/μl), and nerve conduction studies showed demyelinating sensorimotor polyneuropathy. The patient developed somnolence and a left extensor plantar response on the 8th day. A diagnosis of Bickerstaff’s brainstem encephalitis was made. Treatment with plasmapheresis led to a rapid improvement of clinical symptoms. To date, only five similar cases have been reported, but this is the only case in which PRES developed prior to treatment.

**Conclusions:**

PRES can be a comorbid condition with Bickerstaff’s brainstem encephalitis, either preceding or following treatment; caution should be used in patients with either syndrome who exhibit atypical presentations.

## Background

Posterior reversible encephalopathy syndrome (PRES) is characterized by headache, confusion, seizures and visual loss with different triggers and associated conditions, such as acute hypertension, acute kidney injury, eclampsia, sepsis, multi-organ failure, and autoimmune disease [1]. The typical neuroimaging findings are reversible vasogenic subcortical edema involving the posterior hemispheres bilaterally. Some case reports have demonstrated the co-occurrence of Guillain-Barre syndrome (GBS) (or its spectral disorder) and PRES [2], and intravenous immunoglobulin (IVIG) has been implicated as the cause of PRES in a few cases [3–7]. The incidence of GBS in the general population is 0.75–2 cases per 100,000 [8]. The actual incidence of PRES is unknown, but it is estimated to be rare. Therefore, the co-occurrence of both syndromes is unlikely by chance, but rather implicates a common pathogenic basis.

Here we report a patient in whom PRES was the presenting manifestation of Bickerstaff’s brainstem encephalitis (BBE) and discuss the possible pathophysiological mechanisms.

## Case presentation

**Fig. 1:**
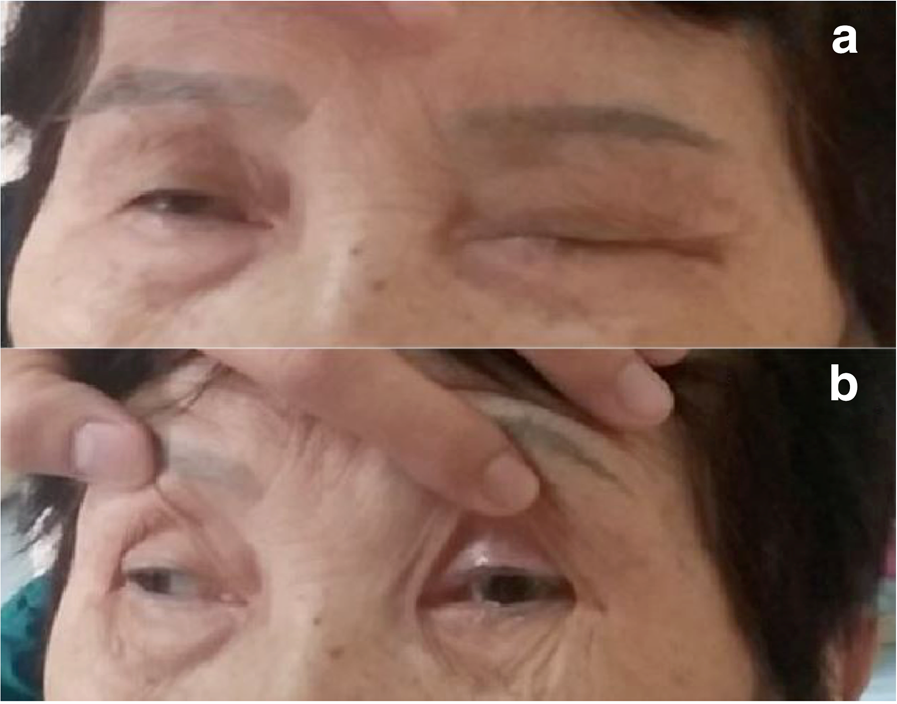
Clinical pictures of our patient on the 5^th^ day. **a** bilateral ptosis. **b** left eye impaired adduction on right gaze

**Fig. 2:**
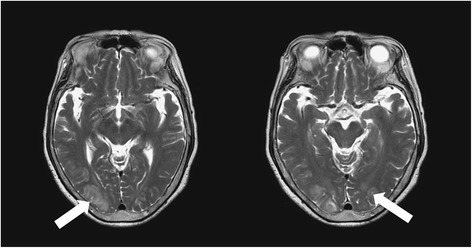
Brain MRI of our patent on the 4^th^ day. Brain MRI T2-weighted hyper-intense signal abnormalities in the occipital lobes bilaterally

A previously healthy 75-year-old woman complained of severe headache of the bilateral frontal area, accompanied by blurred vision and mild left eyelid ptosis for 3 days. She had upper respiratory infection symptoms (cough and sneezing) 2 weeks prior to presentation. She developed arterial hypertension (210/100 mmHg) at the emergency room (on the 4th day after symptom onset). Neurological examination showed left eyelid ptosis and limited extraocular movements horizontally in the left eye with the pupil sparing (Fig. 1). There were no sympathetic signs, such as miosis and anhydrosis, nor any long tract signs. A computed tomography-angiography (CTA) excluded cerebral aneurysm or other compressive lesions of the oculomotor nerve. Brain magnetic resonance imaging (MRI) showed T2-weighted hyper-intense signal abnormalities in the occipital lobes bilaterally (Fig. 2), appearing iso-intense on diffusion-weighted imaging, with an increase of the apparent diffusion coefficient, consistent with vasogenic edema. These findings were compatible with the diagnosis of PRES. Because of persistent headache and elevated systolic blood pressure, amlodipine and olmesartan were administered. However, the conditions did not improve until nimodipine, 30 mg 4 times a day, was added. On the next day, partial ptosis of the right eye developed, followed by bilateral oculomotor and trochlear nerve palsy. Generalized hyporeflexia with bilateral flexor plantar responses was noted. The Romberg test was positive. The finger-nose-finger and heel-knee-shin tests were bilaterally impaired, and an ataxic gait was present. On the 6th day, the patient experienced weakness of the lower limbs bilaterally (MRC: 3/5) and could no longer stand. The partial ptosis of the right eyelid progressed to complete ptosis, and both eyes showed complete external ophthalmoplegia. A test for acetylcholine receptor antibody was negative. Additional laboratory studies, including erythrocyte sedimentation rate, complement factors C3 and C4, antinuclear antibodies, extractable nuclear antigens, antineutrophil cytoplasmic antibody, RNP, anti-SSA, anti-SSB, anti-dsDNA, rheumatic factor, thyroid function, estrogen, FSH, LH, cortisol, ACTH, prolactin, HbA1C, hepatic, and renal function, were within normal limits. Antibodies against gangliosides (including GQ1b) were not checked because of unavailability. Cerebrospinal fluid examination demonstrated albumin-cytological dissociation (protein: 66.6 mg/dl, white cell count: 0/μl, glucose: 66 mg/dl (serum glucose: 114 mg/dl) and albumin: 39.69 mg/dl) and an elevated IgG index (0.84). A panel of CSF analysis, including cytology, bacterial culture, fungal culture, and serological titers of viruses including CMV, HSV1, and HSV2, etc. all showed negative results. A nerve conduction study revealed loss of sensory nerve potentials of the bilateral sural, medial, and ulnar nerves. Motor nerve conduction studies showed demyelinating and eventually axon sensorimotor polyneuropathy (Table 1), which compatible with acquired demyelination neuropathy. Needle electromyography was unrevealing. On the 8th day, the patient had altered consciousness (Glasgow coma scale was E3V4M5) and an extensor plantar response on the left. Hypoxia, uremia, Wernicke’s encephalopathy, Addison’s disease, hepatic encephalopathy, and alcoholism were excluded on the basis of physical and laboratory findings; however, euvolemic hyponatremia (Na:119 mEq/L), compatible with syndrome of inappropriate antidiuretic hormone secretion (SIADH), was noted. Other co-morbidities for hyponatremia or SIADH including malignancy, intracranial infection, hemorrhage, lung disease…etc., have been excluded in our patient. A diagnosis of Bickerstaff’s brainstem encephalitis with overlapping GBS and SIADH was made. Alternate day plasmapheresis was instituted for five times and the hyponatremia was gradually corrected. Her consciousness level and ophthalmoplegia improved gradually, and she could open her eyes on the 13th day. The patient received an intensive rehabilitation program and showed progressive improvement of neurological deficits. She could walk with cane and showed mild lower limb weakness (MRC: 4/5) on the 17th day. She was discharged on the 21st day with residual mild weakness and dysesthesia of the lower extremities. Two months later, she had completely recovered.

**Table 1:** Motor nerve conduction study. CMAP: compound muscle action potential

Table 1.	Distant latency (ms)	Conduction velocity (m/s)	CMAP (mV)
L. median n.	5.9	26.8	3.0
R. median n.	NA	NA	NA
L. ulnar n.	4.6	43.1	3.3
R. ulnar n	5.0	44.7	3.3
L. peroneal n.	6.3	29.9	2.1
R. peroneal n.	5.7	31.3	3.6
L. tibial n.	5.9	28.9	3.6
R. tibial n.	6.9	31.7	6.5

## Conclusions

Our patient presented a clinical and neuroradiological pattern characteristic of PRES preceding a constellation of neurological symptoms, including complete ptosis, external ophthalmoplegia, progressive ataxia, weakness of the lower limbs bilaterally, altered consciousness, and a left extensor plantar reflex, which were consistent with Bickerstaff’s brainstem encephalitis with overlapping GBS [9]. Although previous reports have suggested that PRES could be the presenting manifestation of GBS [10, 11] and could occur in patients with GBS variants, such as Miller-Fisher syndrome (MFS) or BBE (Table 2), our case, in contrast to most of the cases, presented with PRES as the first manifestation of BBE with overlapping GBS prior to any intervention. Three previous cases have shown the co-occurrence of MFS or MFS/ BBE-overlap syndrome and non-hypertensive PRES; however, in these cases, IVIG treatment was considered to be the culprit of PRES [4, 5, 12]. One recent case demonstrated extensive vasogenic edema in BBE without any intervention; however, whether the vasogenic edema of the deep white matter, brainstem, and cerebellum was attributable to PRES is uncertain [13].

**Table 2:** Four cases of clinical manifestation of MFS/BBE associated with PRES

Age (y/o)	Sex (M/F)	Initial GBS (MFS/BBE) symptoms and signs				Initial PRES symptoms	HTN	MRI findings	antecedent infection	IVIG intervention	
		OAA	Mental disturbance	Weakness	Plantar reflex						
53	M	+	NA	−	NA	Headache (4 days after IVIG)	200/130 mmHg (4 days after IVIG)	Bilateral O-P-T Cerebellum Basal ganglion Brainstem	Yes	Yes	[4]
29	F	+	+ (48 h after IVIG)	+	NA	Altered mental status (48 h after IVIG)	−	Brain stem, Bilateral F-P-O	No	Yes	[5]
54	F	+	+ (24–48 h after IVIG)	−	bilateral flexor	Headache Seizure (24–48 h after IVIG)	−	Bilaterally P-O	No	Yes	[10]
75 (our patient)	F	+	+ (day 8)	+ (day 6)	left extensor	Headache Blurred vision	210/100 mmHg (day 4)	Bilateral O	Yes URI: 14 days before symptoms onset	No	

*Abbreviations*: *MFS* Miller-Fisher syndrome, *BEE* Bickerstaff brainstem encephalitis, *PRES* posterior reversible encephalopathy syndrome, *OAA* ophthalmoplegia, ataxia, and areflexia, *HTN* hypertension, *URI* upper respiratory infection, *O* occipital lobe, *P* parietal lobe, *T* temporal lobe, *F* frontal lobe

The causal relationship between PRES and BBE is uncertain, but presumably is similar to that between PRES and GBS. There are some possible mechanisms that might explain the association between GBS and PRES [14]. One of them is dysautonomia. Dysautonomia is reported in 52 % to two thirds of all GBS patients [15, 16]. It can lead to a marked blood pressure surge with overwhelmed cerebrovascular auto-regulation, causing increased brain-blood capillary permeability, impaired blood-brain barrier, and eventually the development of PRES [17]. The other one is the increased level of circulating cytokines and chemokines. Pro‐inflammatory cytokines, such as interferon‐γ and tumor necrosis factor‐α, released by T lymphocytes play a critical role in the pathogenesis of inflammatory demyelination of the peripheral nervous system [18]. Increased levels of chemokines, such as CCL2-CCR2, CCL5-CCR5, and CXCL10-CXCR3, have been found in GBS and experimental autoimmune neuritis in humans and animal studies, respectively [19, 20]. These pro-inflammatory mediators may also contribute to the pathogenesis of PRES by changing capillary permeability and by enhancing the disruption of the blood-brain barrier [21].

The etiology of SIADH in our patient was uncertain, but we considered it to be comorbidity attributed to her Bickerstaff’s brainstem encephalitis. SIADH has been reported in 7–48 % of patients with GBS [22, 23]. The mechanism of which was hypothesized to be downward osmotic resetting and enhanced renal tubular sensitivity to antidiuretic hormone [24–26]. Of note, SIADH has been associated with poor outcome in GBS as bulbar weakness and such as the need of ventilator support or longer hospitalization period [22]. Fortunately, our patient did not run into this devastating disease cause.

To conclude, PRES could be the initial presentation of BBE, either due to an immunological reaction against both the central and peripheral nervous systems or as a result of acute hypertension caused by autonomic dysfunction. Although the accurate rate of co-occurrence of these two syndromes remains to be explored in large-scale prospective studies, a comorbid PRES should be considered in patients with initial signs of GBS or its spectral disorders, especially in those with a blood pressure surge or visual field defect.

## Abbreviations

BBE: Bickerstaff brainstem encephalitis
GBS: Guillain-Barre syndrome
IVIG: Intravenous immunoglobulin
MFS: Miller-Fisher syndrome
PRES: Posterior reversible encephalopathy syndrome
SIADH: Syndrome of inappropriate antidiuretic hormone secretion
